# Physical Exercise Promotes Novel Object Recognition Memory in Spontaneously Hypertensive Rats after Ischemic Stroke by Promoting Neural Plasticity in the Entorhinal Cortex

**DOI:** 10.3389/fnbeh.2017.00185

**Published:** 2017-11-08

**Authors:** Xiaona Pan, Ting Jiang, Liying Zhang, Haiqing Zheng, Jing Luo, Xiquan Hu

**Affiliations:** Department of Rehabilitation Medicine, Third Affiliated Hospital of Sun Yat-sen University, Guangzhou, China

**Keywords:** physical exercise, novel object recognition (NOR), transient middle cerebral artery occlusion (tMCAO), entorhinal cortex, neural plasticity

## Abstract

Cerebral ischemia leads to memory impairment, and several studies have indicated that physical exercise (PE) has memory-improving effects after ischemia. This study was designed to further explore the specific role of PE in novel object recognition (NOR) memory after stroke and the exact cortical regions in which memory is restored by PE. Spontaneously hypertensive rats (SHR) were subjected to transient middle cerebral artery occlusion (tMCAO) or sham surgery, followed by 26 days of PE starting on day 3 post-tMCAO. Thereafter, infarct volume, neurobehavioral outcome and NOR memory were assessed. Immunofluorescence staining and Luxol Fast Blue (LFB) staining were performed in the prefrontal cortex, entorhinal cortex and corpus callosum regions. Western blot analysis was performed to detect expressions of Nestin, Bcl-2 and SYN proteins in the entorhinal cortex. After tMCAO, NOR memory impairment was found in SHR. Rats subjected to PE post-tMCAO showed increased discrimination ratio, as well as significant decreases in infarct volumes and modified neurological severity scores (mNSS), when compared with tMCAO rats without PE. After stroke, NeuN-positive cell number was drastically reduced in the entorhinal cortex, rather than in the prefrontal cortex. Ischemic stroke had no impact on myelin and phospholipids, and the ratio of SMI-32/MBP in the corpus callosum. PE increased NeuN, Nestin, Ki67, MBP, SYN, PSD-95 and Bcl-2 expressions in the entorhinal cortex, while TUNEL and SMI-32 expressions were decreased. In conclusion, the NOR memory-improving capacity promoted by PE was closely related to neuronal cell proliferation and synaptic plasticity of the entorhinal cortex.

## Introduction

The novel object recognition (NOR) test, which is a non-reinforcement based paradigm that relies on spontaneous exploratory behavior, is widely used to study nonspatial learning and memory in animals (Lucon-Xiccato and Dadda, 2016). It was first described in 1988 by Ennaceur and Delacour (1988). The function of object recognition memory in rodents, in certain degree, is similar to the face identification or scene reconstruction in human (Cohen and Stackman, 2015). Recent studies have shown that NOR tests can be adapted for use in non-rodent species, such as monkeys (Zola et al., 2000; Malkova and Mishkin, 2003), horses (Visser et al., 2002) and pigs (Gifford et al., 2007). Moreover, this test has also been used to investigate the effects of various pharmacological treatments on types of brain damage, including stroke (Goulart et al., 2010; Samson et al., 2015).

Ischemic stroke remains to be one of the most serious neurological diseases, which results in permanent deterioration in motor and cognitive abilities. The memory impairment induced by stroke can be divided into spatial and nonspatial cognitive disorders. Over the past decades, clinical and technological advances brings about a relative lower mortality at the early stage of ischemic stroke than before, however, cognitive dysfunctions still play significant adverse effects on the quality of daily life for patients who are suffered with ischemic stroke (Knecht et al., 2011). In addition to spatial memory, NOR memory is another important component of cognition.

Physical exercise (PE) has been characterized as a positive health behavior providing physiological benefits, such as obesity prevention (Sakurai et al., 2017), cardiovascular protection (Broderick et al., 2017) and even resistance against certain cancers (Kim et al., 2017). Previous studies have also demonstrated that PE is beneficial in ischemic brain recovery, by reducing brain infarct volume and improving neurological outcomes (Yang et al., 2003). In addition, PE can alleviate ischemia-related cognitive learning and memory deficits (Schimidt et al., 2014). For instance, treadmill exercise improved cerebral ischemia-induced impairment of short-term memory and spatial working memory (Seo et al., 2014). At the same time, there are few studies reported the effect of exercise training on the nonspatial learning and memory of rodents after stroke. It has been proposed that the prefrontal lobe, entorhinal cortex and corpus callosum are involved in nonspatial learning and memory processes (Yee and Rawlins, 1998; Granon and Poucet, 2000; Blasi et al., 2014; Brown et al., 2016). However, the exact brain regions and mechanisms by which PE impacts stroke-induced nonspatial learning and memory deficit are still unclear.

In the present study, the transient middle cerebral artery occlusion (tMCAO) model was established in spontaneously hypertensive rats (SHR) as an *in vivo* model of focal cerebral ischemia. Starting on day 3 post-tMCAO, rats were subjected to 26 days of PE; thereafter, the changes in infarct volume, neurobehavioral outcomes and NOR memory were assessed. In addition, the exact cortical regions involved in the mechanism by which PE improves recovery from tMCAO-induced memory impairment were investigated. These results will help elucidate the specific role of PE in recovery from stroke-induced nonspatial learning and memory impairment.

## Materials and Methods

### Animals

A total of 90 male SHR (aged 10–12 weeks, weight, 250–280 g) were obtained from the Experimental Animal Center of Sun Yat-sen University (Guangzhou, China). All the animal experiments performed in this study were approved by the Animal Ethics Guidelines for the Care and Use of Laboratory Animals of the National Institute of Health (Publication No. 80-23, revised 1996).

### Study Design

SHR were randomly divided into four groups (*n* = 20 rats/group): (1) a Sham group that received sham surgery; (2) a tMCAO (2 d) group, in which rats underwent tMCAO surgery and NOR tests were performed 2 days later; (3) a tMCAO (28 d) group, in which NOR tests were performed at 28 days post-tMCAO; and (4) a tMCAO (28 d) + PE group, in which tMCAO was established and rats exercised in a running wheel for 26 consecutive days starting at the third day post-tMCAO. Besides, 10 rats were received sham surgery and exercised in a running wheel for 26 consecutive days starting at the third day post-surgery, as a Sham + PE group.

### tMCAO

SHR were anesthetized by intraperitoneal (IP) injection of chloralhydrate (350 mg/kg), and tMCAO was then performed as previously described (Zhang et al., 2013). In brief, a 4-0 nylon suture filament with a rounded tip was inserted into the internal carotid artery to block the middle cerebral artery at its origin. After 90 min, the filament was removed to allow reperfusion. Body temperature was maintained at 37 ± 0.5°C by a heat lamp (FHC, Bowdoinham, ME, USA) throughout the surgery. The rats in the Sham group underwent sham surgery without suture insertion.

### PE

Rats subjected to running wheel exercise plan were placed into a programmable, motorized wheel apparatus (21 cm diameter, 40 cm length; South China University of Technology, Guangzhou, China), permitting the quantification of exercise intensity. Rats in the PE group were placed into the wheel and subjected to a period of exercise for 26 days starting at day 3 post-tMCAO. During the first 12 days, the running speed was set at 5 rev/min (approximately 3 m/min), for 20 min, twice a day (8 a.m. and 8 p.m.). For the next 14 days, the running speed was increased to 10 rev/min (approximately 6 m/min). The rats in the Sham and tMCAO groups were housed in a standard cage with free access to food and water, with no specific exercise plan.

### 2,3,5-Triphenyltetrazolium Chloride (TTC) Staining

The infarct volume of SHR in each group (*n* = 5) was assessed using 2,3,5-Triphenyltetrazolium chloride (TTC) staining (Wang et al., 2016). Briefly, the brains of SHR from Bregma +4.0 mm to −6.0 mm were sliced into six 2.0-mm-thick sections. After incubation in 2% TTC solution (Sigma, St. Louis, MO, USA) for 30 min at 37°C, coronal brain sections were then fixed in 4% paraformaldehyde solution overnight. The infarct area was identified by non-staining region while the live area should turn red. Image analysis was carried out independently by two observers (both XP and LZ with 4 years of experiences). The volume of infarction was measured with Image-Pro Plus software (Media Cybernetics, Silver Spring, MD, USA). The relative infarct volume was manually calculated according to the following formula: infarct percentage = (volume of the contralateral hemisphere − volume of the non-infarct contralateral hemisphere)/volume of the contralateral hemisphere × 100%.

### Modified Neurological Severity Score (mNSS)

The modified neurological severity scores (mNSS) was recorded at 2, 14 and 28 days post-tMCAO as described previously (Liu et al., 2011). The mNSS is a composite of the motor, sensory, balance and reflex tests and is assessed on a scale of 0–18 (normal score 0, maximal deficit score 18). Rats were tested three times by an individual blinded to grouping, and the average was recorded.

### NOR Tests

NOR tests were conducted in an open box (72 × 72 × 35 cm) with clear plastic walls and a black plastic floor. The box was placed in a dark room under infrared light throughout the test. On the day before the NOR test, rats were allowed to explore the empty box freely for 1 h. In the first trial, two identical objects (A and B) were placed in the upper right and lower left quadrants of the box, and placed rats into the box. After 10 min of exploration, rats were removed from the box and allowed a 1 h break. In the second trial, object B was replaced with object C (with C being dissimilar to both A and B). The rats were then put back into the box for a further 5 min period of exploration. The behavior of rats was video recorded and the evaluation of NOR memory was expressed as a percentage of the discrimination ratio calculated according to the following formula: Discrimination ratio (%) = (*N* − *F*)/(*N* + *F*) × 100%, where *N* represents the time spent in exploring the new object and *F* represents the time spent in exploring the same object.

### Tissue Preparation for Histochemistry

On days 2 and 28 post-tMCAO, rats (*n* = 10) were anesthetized with 1 g/kg chloralhydrate and perfused transcardially with 150 mL normal saline (4°C), followed by 200 mL of 4% paraformaldehyde (4°C; Zheng et al., 2014). The rats from sham group (*n* = 10) were subjected to the same steps. The brains were then removed and fixed in 4% paraformaldehyde for 8 h at 4°C, prior to sequential immersion in 20% and 30% sucrose until the tissue no longer floated in the solution. Coronal sections were cut on a cryostat (CM1900; Leica, Heidelberger, Germany). The sections of the prefrontal cortex from the Bregma +4.20 to +2.20, the corpus callosum from the Bregma 0.20 mm to −2.20 mm and the entorhinal cortex from the Bregma −4.80 to −7.04 were processed for immunofluorescence staining. Coronal sections (30 μm thick) were processed for Luxol Fast Blue (LFB) staining.

### Immunofluorescence Staining

Immunofluorescence staining of NeuN, Nestin, Ki67, MBP, SMI-32, SYN and PSD-95 was performed using previously reported methods (Zheng et al., 2014). Briefly, sections were pre-treated with citrate buffer (0.01 mol/L, pH 6.0) for 5 min at 85°C, and then incubated in 5% normal goat serum for 1 h at room temperature. Subsequently, the sections were incubated overnight at 4°C with antibodies for the detection of mouse anti-NeuN (1:200; Millipore, Billerica, MA, USA), mouse anti-Nestin (1:200; Millipore), rabbit anti-Ki67 (1:500; Abcam, Cambridge, MA, USA), rabbit anti-MBP (1:500, Abcam), mouse anti-SMI-32 (1:500, Calbiochem, NE1023), mouse anti-SYN (1:100; Abcam) and rabbit anti-PSD-95 (1:1000, Abcam). After washing three times with phosphate-buffered saline (PBS), the sections were incubated with the appropriate peroxidase-conjugated secondary antibodies anti-mouse IgG (1:1000, Cell Signaling Technology, Danvers, MA, USA) and anti-rabbit IgG (1:1000, Cell Signaling Technology) for 1 h at room temperature. Fluorescence signals were observed with an optical microscope (BX51; Olympus, Tokyo, Japan).

All histological images captured using the same exposure conditions were analyzed with the Image-Pro Plus analysis software (Media Cybernetics, Silver Spring, MD, USA). Eight consecutive sections of the prefrontal cortex, corpus callosum and entorhinal cortex were analyzed. The cell counts in four non-overlapping fields (425 μm × 320 μm) under an optical magnification of 400× were recorded and presented as the average cell number per field for each section.

### LFB and Nissl Staining

The sections were first immersed in xylene followed by anhydrous ethanol gradient dehydration. Thereafter, the sections were stained overnight in 0.1% LFB solution (Sigma-Aldrich, St. Louis, MO, USA) at 60°C. After washing with 95% ethyl alcohol and distilled water, the sections were differentiated in 70% ethyl alcohol for 30 s. Differentiation was terminated by washing in distilled water until the unmyelinated tissue appeared white. For Nissl staining, the sections were stained with cresyl violet to assess changes in the number of Nissl bodies.

### Terminal Deoxynucleotidyl (TUNEL) Assay

Deoxynucleotidyl Transferase-Mediated dUTP *in situ* Nick-End Labeling (TUNEL) assay was performed in the entorhinal cortex region, using an *in situ* cell death detection kit (Fluorescein; Roche Corp., Basel, Switzerland). TUNEL signals were detected with an optical microscope (BX51; Olympus). Visible TUNEL-positive cells were counted by Image-Pro Plus image analysis software in each group.

### Western Blot Analysis

The remaining SHR (*n* = 5) were deep narcotized in each group and perfused intracardially with 50 mL ice-cold 0.9% saline. Then the entorhinal cortex of brain tissue (from Bregma −4.80 to −7.04) was rapidly dissected and homogenized in protein extraction buffer (Thermo, Pierce Biotechnology, Waltham, MA, USA) containing a complete protease inhibitor cocktail (Thermo). Protein concentration was measured with the Bradford Protein Assay (Thermo). Equal amounts of the protein samples were separated by 10% sodium dodecyl sulfate-polyacrylamide gel electrophoresis (SDS-PAGE) and transferred onto polyvinylidene difluoride (PVDF) membranes. After blocking with 5% non-fat dried milk for 1 h at room temperature, the membranes were incubated with rabbit anti-nestin (1:200; Millipore), mouse anti-synaptophysin (1:100, Abcam) rabbit anti-Bcl-2 (1:1000; Cell Signaling Technology) and mouse anti-β-actin (1:1000; Santa Cruz) overnight at 4°C. The membranes were then incubated with the appropriate secondary antibodies (1:1000; Cell Signaling Technology) for 2 h at room temperature. Positive signals were detected by enhanced chemiluminescence (ECL, Cell Signaling Technology) and visualized by exposure to X-ray film.

### Statistical Analysis

The results were presented as the mean ± SEM. Statistical analysis was performed by using SPSS version 13.0 (SPSS Inc., Chicago, IL, USA). Statistical differences of mNSS in different groups were analyzed by the repeated measures ANOVA. All other statistical differences were analyzed by ANOVA with a LSD-t procedure. *P*-values < 0.05 were considered to indicate statistical significance.

## Results

### PE Reduces Brain Infarct Volume and Improves Neurobehavioral Outcome in tMCAO Rats

**Figure 1:**
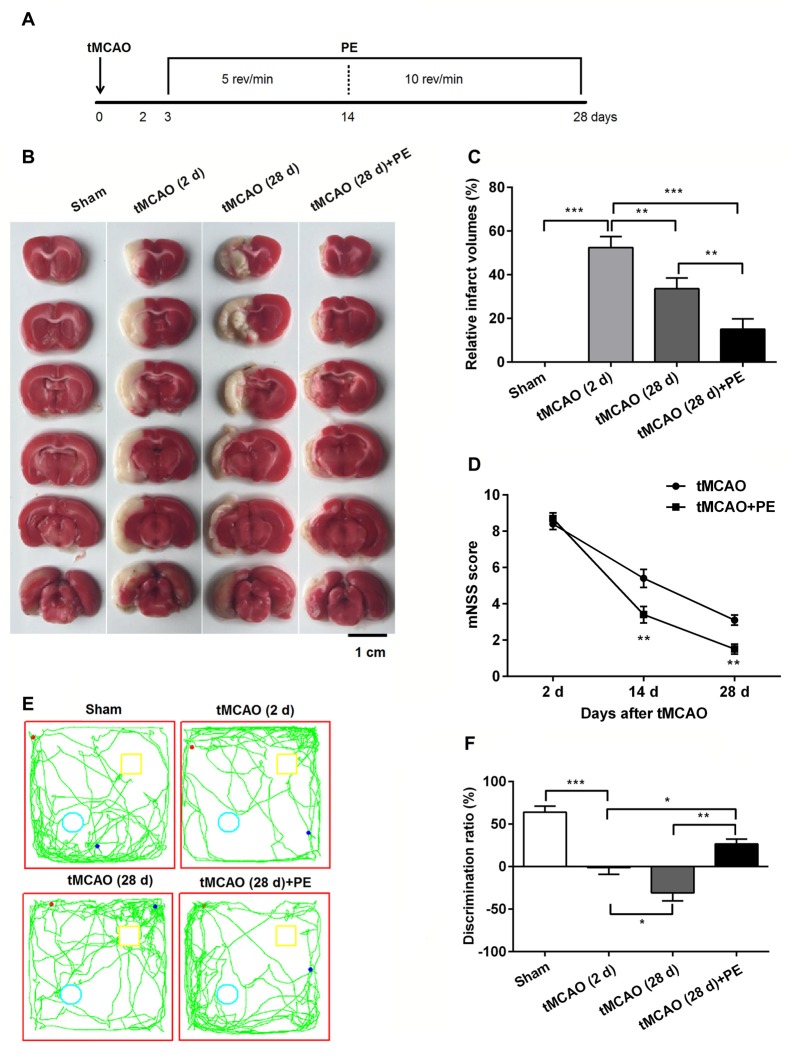
Physical exercise (PE) improves novel object recognition (NOR) memory in transient middle cerebral artery occlusion (tMCAO) rats. **(A)** Spontaneously hypertensive rats (SHR) were subjected to tMCAO or sham surgery, followed by 26 days of PE starting on day 3 post-tMCAO. Thereafter, infarct volumes were **(B)** imaged and **(C)** measured *n* = 5; **(D)** neurobehavioral outcomes were evaluated based on modified neurological severity scores (mNSS) *n* = 20; **(E)** Exploration patterns were video recorded; green lines are exploring patterns of rats, red dots represent the points where rats were placed in the test room, pale blue circle and yellow square represent two different objects. **(F)** Discrimination ratios were measured. *n* = 10; *P < 0.05; **P < 0.01; ***P < 0.001.

Previous research has emphasized that tMCAO induces brain infarction and neurobehavioral function impairment (Lourbopoulos et al., 2010; Wang et al., 2015); this was also confirmed in our study (Figure 1). Obvious brain infarcts were observed 2 days post-tMCAO. Subsequently, the infarct volumes decreased, with minimum infarct volumes detected at day 28 post-tMCAO (*P* < 0.001, Figures 1B,C). Rats at day 2 post-tMCAO showed an extremely high mNSS (8.4 ± 1.0), and the score was decreased to 5.4 ± 1.6 at day 14 and to 3.1 ± 0.9 at day 28 (Figure 1D). The repeated measures ANOVA revealed a significant main effect of (mNSS of tMCAO + PE group < mNSS of tMCAO group, *F*_(1,9)_ = 6.076, *P* = 0.024), an significant interaction between treatment effects and time effects (*F*_(2,36)_ = 16.051, *P* < 0.001) and a significant time effect (*F*_(2,36)_ = 430.122, *P* < 0.001). Of note, rats in the tMCAO + PE group showed significant decreases in infarct volumes and mNSS when compared with rats in the tMCAO group without PE (*P* < 0.05 and *P* < 0.01, respectively), which indicated that PE is beneficial in neurological function recovery after brain infarction.

### PE Improves NOR Memory Impairment after tMCAO in SHR

Next, we investigated the ability of PE to improve the recovery of NOR memory in tMCAO rats. No significant difference of discrimination ratio was observed between Sham and Sham + PE groups (*P* > 0.05, Supplementary Figure S1). As shown in Figures 1E,F, the discrimination ratio was decreased at day 2 post-tMCAO, and reached a minimum 28 days later (*F* = 27.39, *P* < 0.001). At day 28 post-tMCAO, rats subjected to PE exhibited a much higher discrimination ratio than that of rats subjected to tMCAO without PE (26.6 ± 16.9% vs. −31.0 ± 28.4%; *P* < 0.01). These data indicated that ischemic stroke was related with the decline of NOR memory and PE improves NOR memory in tMCAO rats.

### PE Improves Recovery from Entorhinal Cortex Injury Induced by tMCAO

**Figure 2:**
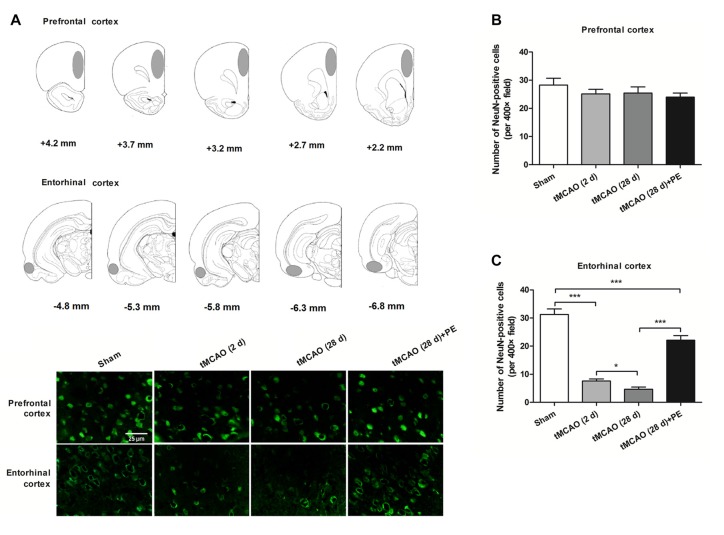
PE improves recovery from entorhinal cortex injury induced by tMCAO. After tMCAO rats were subjected to PE, the brains were removed and the sections of the prefrontal cortex and the entorhinal cortex were processed for **(A)** immunofluorescence staining of NeuN. **(B,C)** Numbers of NeuN-positive cells based on immunofluorescence staining analysis. *n* = 10; *P < 0.05; ***P < 0.001.

Increasing evidence indicates that the prefrontal and entorhinal cortex are closely involved in the neural circuitry of NOR memory (Wilson et al., 2013; Chao et al., 2016). Thus, we examined the neurons in the prefrontal cortex and the entorhinal cortex after stroke to evaluate the impacts of PE on neurogenesis in ischemia-injured brains. In the prefrontal cortex, the NeuN-positive cell numbers in the Sham, tMCAO and tMCAO + PE groups were equivalent (*F* = 1.22, *P* = 0.32, Figures 2A,B). However, the NeuN-positive cell numbers were significantly reduced in the entorhinal cortex post-tMCAO (*P* < 0.001), with minimum numbers observed at day 28 post-tMCAO (*P* < 0.05; Figures 2A,C). The number of NeuN-positive cells in rats subjected to PE was much higher than that in the tMCAO rats without PE (22.1 ± 5.3 vs. 4.7 ± 2.4; *P* < 0.001).

**Figure 3:**
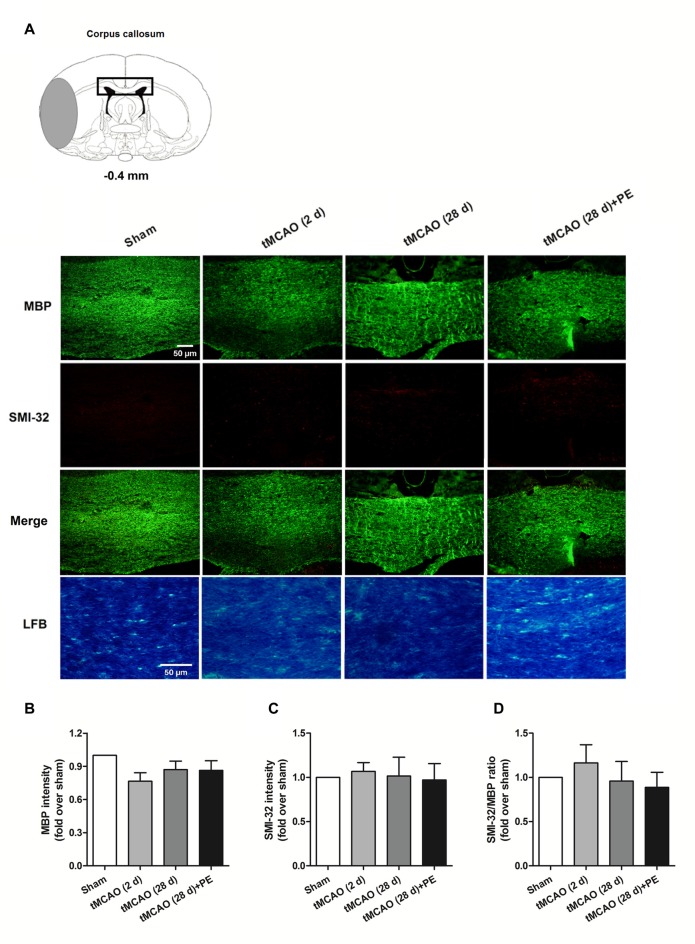
The corpus callosum is not involved in PE mediated recovery from brain injury induced by tMCAO. After tMCAO rats were subjected to PE, the brains were removed and sections of corpus callosum were processed for **(A)** immunofluorescence staining of MBP and SMI-32, as well as Luxol Fast Blue (LFB) staining. Intensities of **(B)** MBP and **(C)** SMI-32, as well as **(D)** the ratio of SMI-32/MBP based on immunofluorescence staining analysis. *n* = 10.

In addition to the prefrontal and entorhinal cortex, the corpus callosum has also been reported to be associated with mild cognitive impairment (Blasi et al., 2014; Brown et al., 2016). Therefore, we performed immunofluorescence staining of MBP (a marker of myelin integrity) and SMI-32 (a marker of abnormally dephosphorylated neurofilament protein) to further evaluate pathological changes in the corpus callosum, as well as LFB staining (a method used to stain myelin and phospholipids) in the corpus callosum. As shown in Figures 3A–D, there were no remarkable differences in the expression of MBP (*F* = 1.938, *P* = 0.143) or SMI-32 (*F* = 0.074, *P* = 0.974) between the Sham, tMCAO and PE + tMCAO groups. Furthermore, no obvious changes in LFB stained myelin and phospholipids were observed in rats post-tMCAO.

### PE Promotes Neuronal Cell Proliferation and Synaptic Plasticity in Entorhinal Cortex

**Figure 4:**
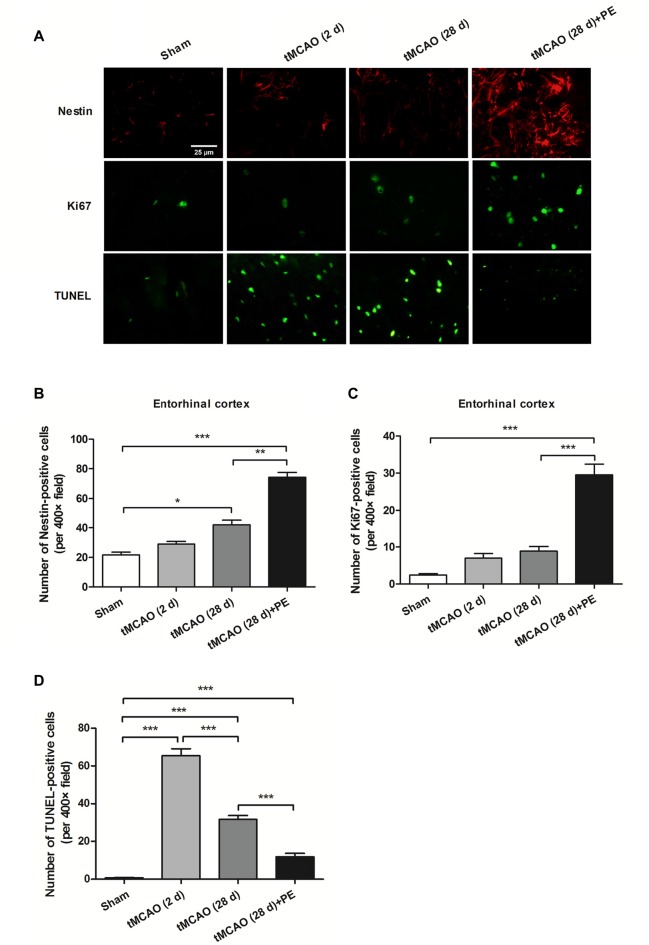
PE promotes neuronal cell proliferation in the entorhinal cortex in tMCAO rats. After tMCAO rats were subjected to PE, the brains were removed and sections of the entorhinal cortex were processed for** (A)** immunofluorescence staining of Nestin and Ki67, and TUNEL-positive cells. Numbers of **(B)** Nestin-, **(C)** Ki-67- and **(D)** TUNEL-positive cells based on immunofluorescence staining analysis. *n* = 10; *P < 0.05; **P < 0.01; ***P < 0.001.

Nissl staining and immunofluorescence staining of NeuN were performed to confirm whether PE recovered ischemic stroke-induced neuronal injury in the entrohinal cortex. As results shown in Supplementary Figure S2, PE slightly increased the numbers of Nissl-stained cells and NeuN-positive cells after sham surgery, and these increases did not reach significant difference (*P* > 0.05). tMCAO groups (2 or 28 d) exhibited decreases in the numbers of Nissl-stained cells and NeuN-positive cells compared with Sham group. As expect, the numbers of Nissl-stained cells and NeuN-positive cells were remarkably increased in tMCAO + PE group, when compared with tMCAO groups (*P* < 0.01 or *P* < 0.001). To further investigate the therapeutic mechanism of PE, we detected the expression of proliferation markers (Nestin and Ki-67) and apoptosis index (TUNEL) in the entorhinal cortex after ischemic stroke (Scholzen and Gerdes, 2000; Endesfelder et al., 2017). As shown in Figures 4A–C, Nestin-positive cell numbers were significantly increased at day 28 post-tMCAO (*P* < 0.05). Ki-67-positive cell numbers were also slightly increased after tMCAO, although this effect did not reach the level of statistical significance. PE dramatically upregulated the expression of Nestin and Ki-67 compared with that in the tMCAO and Sham groups (*P* < 0.01 and *P* < 0.001, respectively). Besides, the number of TUNEL-positive cells was significantly increased after tMCAO (*P* < 0.001) and then restrained by PE (*P* < 0.001; Figures 4A,D).

**Figure 5:**
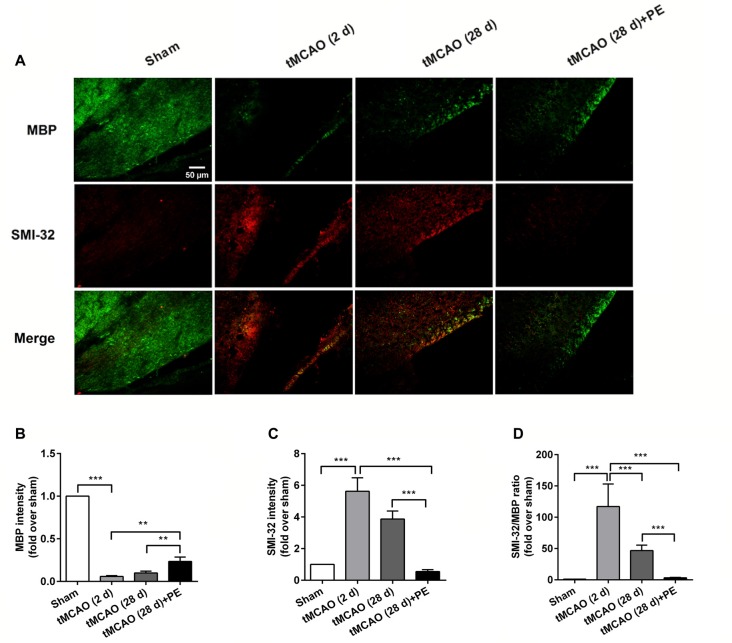
PE promotes the plasticity of neurofilament protein in the entorhinal cortex in tMCAO rats. After tMCAO rats were subjected to PE, the brains were removed and the sections of the entorhinal cortex were processed for **(A)** immunofluorescence staining of MBP and SMI-32. Intensities of **(B)** MBP and **(C)** SMI-32, as well as **(D)** the ratio of SMI-32/MBP based on immunofluorescence staining analysis. *n* = 10. **P < 0.01; ***P < 0.001.

**Figure 6:**
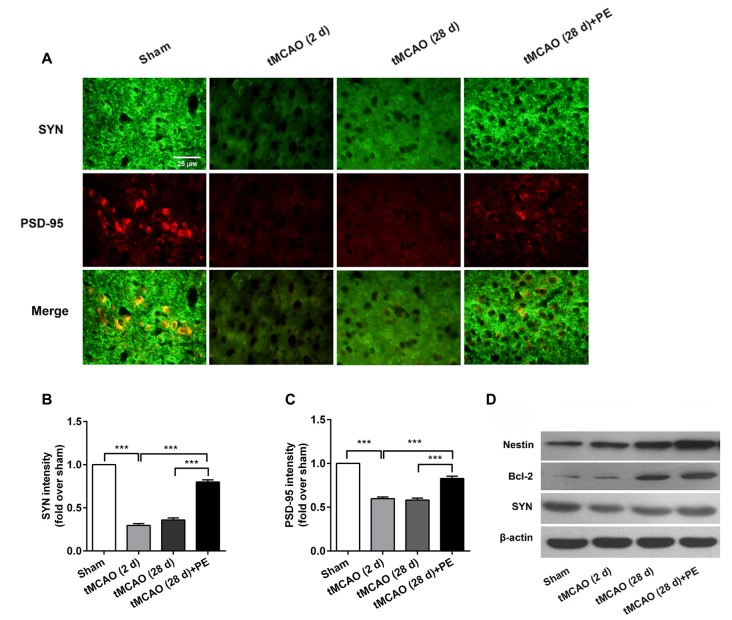
PE promotes neuronal cell proliferation and synaptic plasticity in the entorhinal cortex in tMCAO rats. After tMCAO rats were subjected to PE, the brains were removed and the sections of the entorhinal cortex were processed for **(A)** immunofluorescence staining of SYN and PSD-95. Intensities of **(B)** SYN and **(C)** PSD-95 based on immunofluorescence staining analysis *n* = 10. ***P < 0.001. **(D)** The expression levels of Nestin, Bcl-2 and SYN in the medial temporal lobes were detected by western blotting analysis. β-actin was included as a loading control.

The expression levels of MBP and SMI-32, and two synaptic plasticity-associated proteins (SYN and PSD-95) were also detected in entorhinal cortex. As shown in Figures 5, 6, immunofluorescence staining revealed increased expression of SMI-32 after tMCAO (*P* < 0.001), while expression was decreased after PE intervention (*P* < 0.001). Conversely, expression of MBP, SYN and PSD-95 were all decreased after tMCAO (*P* < 0.001), while expression of all three markers was increased after PE (*P* < 0.01).

Western blotting analysis was performed to assess the expression of Nestin, Bcl-2 and SYN proteins in the entorhinal cortex. As shown in Figure 6D, the western blot results were consistent with those obtained by immunofluorescence staining. The expressions of Nestin and Bcl-2 proteins were remarkably up-regulated by PE after stroke. The expression of SYN was down-regulated in post-tMCAO, but was also up-regulated after PE intervention.

## Discussion

The present study demonstrated that PE post-tMCAO effectively reduced brain infarct volume, improved neurobehavioral outcomes and restored NOR memory in tMCAO rats. tMCAO resulted in marked injury to the entorhinal cortex, rather than the prefrontal lobe or corpus callosum; more importantly, PE improved recovery from tMCAO-induced entorhinal cortex injury. Further investigations revealed that PE promoted neuronal cell proliferation and synaptic plasticity in the entorhinal cortex of SHR. All of these results need further discussion.

Cerebral ischemia leads to many types of memory loss, including impairment of working memory, spatial memory, as well as object recognition memory (Ashabi et al., 2017). It has been widely demonstrated that hypertension is the most important risk factor for ischemic stroke (Gorgui et al., 2014). To investigate the deep pathogenic mechanism of stroke, we established the SHR animal model using rats that suffered from tMCAO. An early study showed that physical activity acted as determinant of functional outcome after cerebral infarction in the rat (Johansson and Ohlsson, 1996). Numerous recent studies have shown that PE has spatial memory-improving effects after ischemia (Schimidt et al., 2014; Seo et al., 2014). In the present study, it was also confirmed that PE post-tMCAO significantly improved the preferences for novel objects in the SHR based on the results of NOR tests. Besides, our data showed that PE has no improving impact on NOR-memory when rats received sham surgery. One explanation could be that memory impairment was not induced by sham surgery, and 26 days of PE is too short to make any significant difference in non-ischemic rats.

Recent studies have shown that the prefrontal and entorhinal cortex and the corpus callosum are three key sites associated with NOR memory function in animals (Wilson et al., 2013; Blasi et al., 2014; Chao et al., 2016). Therefore, in this study, we performed immunofluorescence staining of NeuN, MBP and SMI-32, as well as LFB staining. The results revealed the involvement of entorhinal cortex, rather than the prefrontal lobe or corpus callosum, in PE-induced memory improvements. In addition, numerous researchers suggested that the damage to entorhinal cortex is responsible for the NOR memory loss (Peters et al., 2009; Kebets et al., 2015).

Pathological conditions such as cerebral ischemia increase neurogenesis (Yagita et al., 2001). Liu et al. (1998) demonstrated that cell proliferation in the hippocampal dentate gyrus of gerbils was significantly induced by ischemia. In accordance with these previous studies, we found that the numbers of Nestin- and Ki67-positive cells were significantly increased post-tMCAO, which indicates that neuronal cell proliferation is accelerated by ischemia. More importantly, we found that PE further improved neuronal cell proliferation in the entorhinal cortex after stroke. Similarly, Seo et al. (2014) demonstrated that treadmill exercise alleviated ischemia-induced memory impairment by enhancing cell proliferation and suppressing neuronal apoptosis. We also detected that the expression of TUNNEL was alleviated by PE after stroke.

Synaptic plasticity is important for memory processing (Shih et al., 2013). Evidence of synaptic plasticity impairment following cerebral ischemia provides further insight into the association between brain ischemia and memory deficit (Moghimi et al., 2016). Therefore, in this study we detected the expression of synaptic plasticity-associated proteins to evaluate the role of synaptic plasticity in the mechanism by which PE improves NOR memory in tMCAO rats. Previous studies have shown that synaptic plasticity is enhanced by exercise after ischemia (Nie and Yang, 2017). Additionally, Shih et al. (2013) found that exercise resulted in superior synaptic plasticity and spatial memory performance in tMCAO rats. Our findings are also consistent with these previous reports in that we have demonstrated that PE promotes synaptic plasticity in the entorhinal cortex, which might contribute to nonspatial learning and the formation of NOR memory.

There exist several limitations in the present study. First, the brain injury was unilateral, thus the recovery effects of PE on bilateral injury need to be revealed. Second, we cannot exclude the possibility that the NOR effects in this article are actually related to some other aspect of the ischemic brain injury.

In conclusion, the present study demonstrated that PE improved NOR memory in rats after ischemic stroke. Furthermore, we provide the first evidence that PE induces NOR memory-improvement by promoting neuronal cell proliferation and synaptic plasticity in the entorhinal cortex. These findings implicate that PE, as a non-pharmacological therapeutic strategy for brain ischemic-induced memory impairment, has great potential and broad prospects. However, further studies are required to confirm this hypothesis.

## Author Contributions

In this study, XH designed the work, drafted the manuscript, conducted the experiments and contributed to interpretation of data. XP, LZ and TJ conducted the experiments, analyzed the data and helped to prepare the manuscript. HZ and JL helped to prepare the manuscript and images, collected and analyzed the data and literature. All authors read and approved the final manuscript. All authors agreed to be accountable for all aspects of the study in ensuring that questions related to the accuracy or integrity of any part of the work are appropriately investigated and resolved.

## Conflict of Interest Statement

The authors declare that the research was conducted in the absence of any commercial or financial relationships that could be construed as a potential conflict of interest.

## Supplementary Material

FIGURE S1 Physical exercise (PE) has no significant impact on rats received sham surgery. Spontaneously hypertensive rats (SHR) was subjected to sham surgery, followed by 26 days of PE starting on day 3 post-surgery. **(A)** Exploration patterns were video recorded. **(B)** Discrimination ratios were measured. *n* = 10. ns, no significance.

FIGURE S2 PE increased Nissl-stained cells number and NeuN-positive cells number in transient middle cerebral artery occlusion (tMCAO) rats. **(A)** Nissl staining. **(B)** Immunofluorescence staining of NeuN. **(C)** Quantitative data from Nissl staining. **(D)** Quantitative data from immunofluorescence staining of NeuN. *n* = 10; ns, no significance; ***P* < 0.01; ****P* < 0.001.
