# Supplementary figures and images for: Physical Exercise Promotes Novel Object Recognition Memory in Spontaneously Hypertensive Rats after Ischemic Stroke by Promoting Neural Plasticity in the Entorhinal Cortex
Source: Front Behav Neurosci. 2017 Nov 8;11:185. doi: 10.3389/fnbeh.2017.00185 (PMC5682296; doi:10.3389/fnbeh.2017.00185)

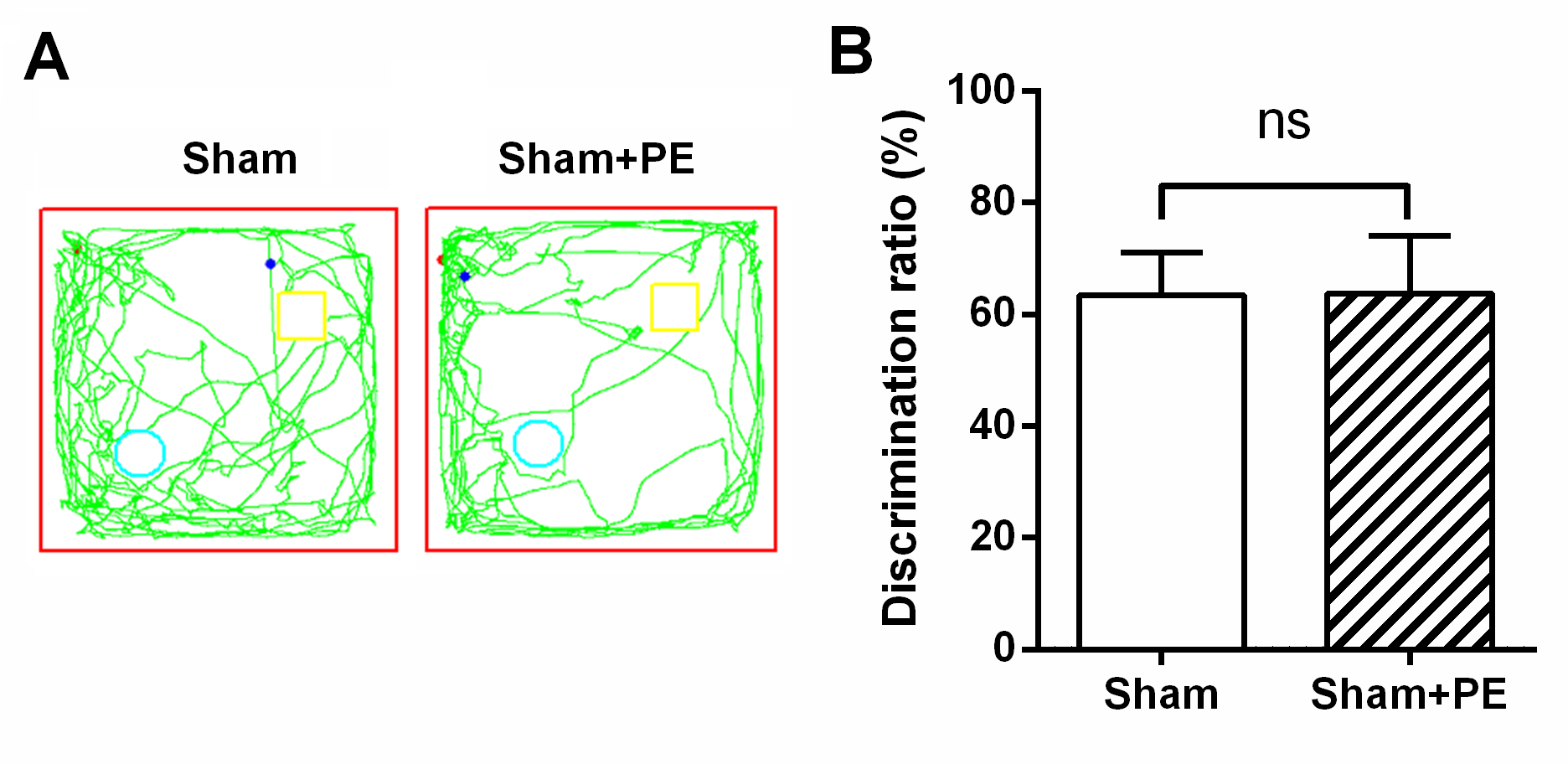

Supplement: FIGURE S1 — Physical exercise (PE) has no significant impact on rats received sham surgery. Spontaneously hypertensive rats (SHR) was subjected to sham surgery, followed by 26 days of PE starting on day 3 post-surgery. (A) Exploration patterns were video recorded. (B) Discrimination ratios were measured. n = 10. ns, no significance. [file Image_1.tif]

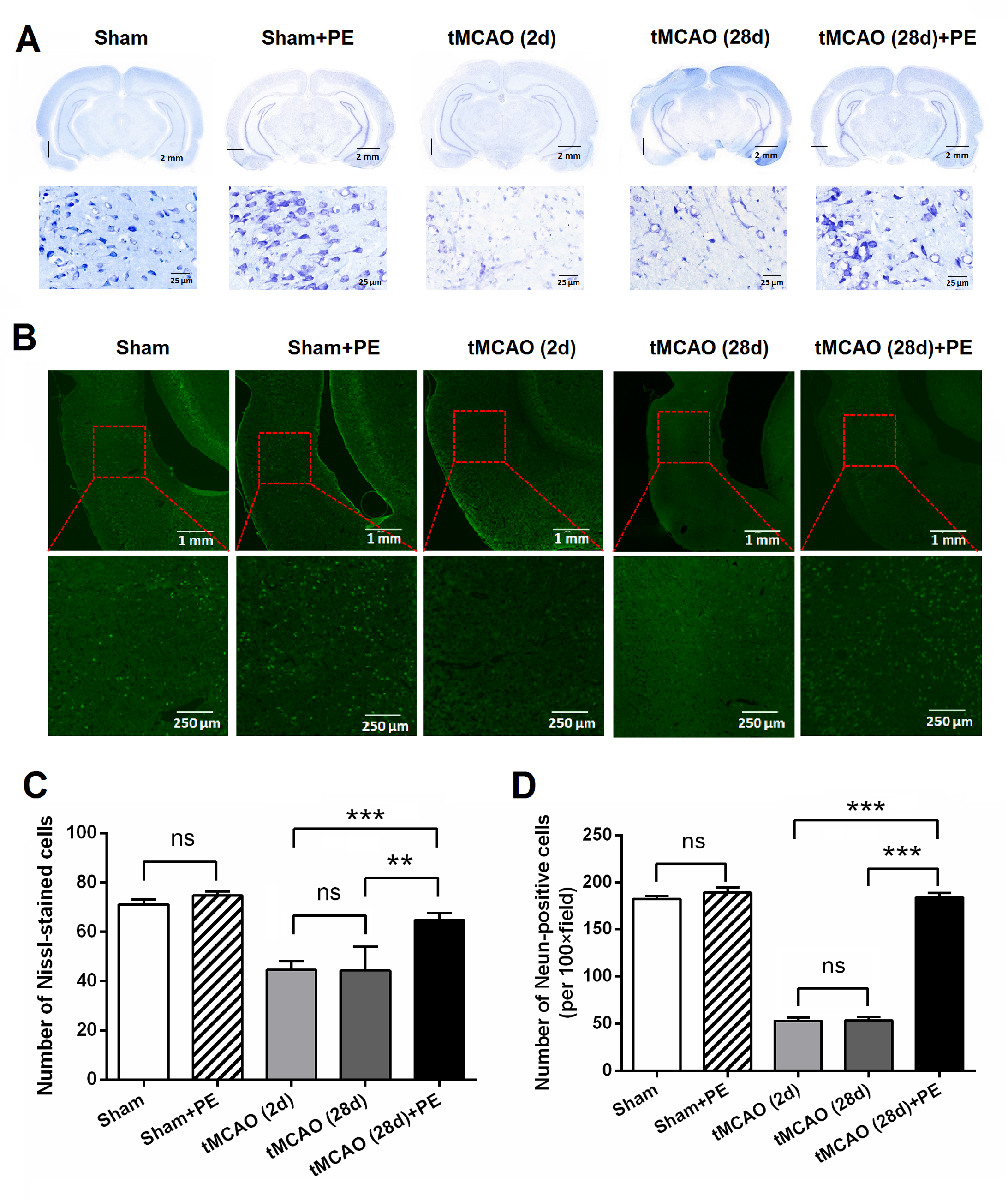

Supplement: FIGURE S2 — PE increased Nissl-stained cells number and NeuN-positive cells number in transient middle cerebral artery occlusion (tMCAO) rats. (A) Nissl staining. (B) Immunofluorescence staining of NeuN. (C) Quantitative data from Nissl staining. (D) Quantitative data from immunofluorescence staining of NeuN. n = 10; ns, no significance; **P < 0.01; ***P < 0.001. [file Image_2.tif]
